# The Perceived Internalized Sexual Stigma Measure (PrISSM): A New Sexual Stigma Measure for Lesbian, Gay, and Bisexual Individuals

**DOI:** 10.3390/bs15091228

**Published:** 2025-09-10

**Authors:** Miguel A. Padilla, Lacey M. Schlappi, Evelyn S. Chiang

**Affiliations:** 1 Department of Psychology, Old Dominion University, 250 Mills Godwin Building, Norfolk, VA 23529, USA; lschl006@odu.edu; 2 Psychology Department, University of North Carolina at Asheville, Asheville, NC 28804, USA; echiang@unca.edu

**Keywords:** internalizes sexual stigma, internalized homophobia, internalized homonegativity, sexual minority health, sexual behavior, LGB, public health, measure, Bayesian methods

## Abstract

Society has long held negative beliefs and attitudes, in the form of sexual stigma, towards sexual minorities (e.g., lesbian, gay, and bisexual; LGB). Sexual stigma can be adopted and internalized by LGB individuals as their own beliefs and attitudes. In LGB individuals, internalized sexual stigma can result in psychological distress such as low self-esteem, depression, shame, and feelings of rejection. However, an instrument to assess internalized sexual stigma specifically developed for LGB individuals is lacking. The present study focuses on the development of a new instrument, the Perceived Internalized Sexual Stigma Measure (PrISSM), that is inclusive of LGB individuals who are 18 years and older. Exploratory and Bayesian confirmatory factor analyses indicate that internalized sexual stigma, as measured by the PrISSM, consists of two factors: internal conflict and disclosure conflict. The two-factor structure is also invariant to LGB individuals. As such, items of the PrISSM do not need to be separately reworded for lesbian, gay, or bisexual individuals. In addition, the PrISSM is a brief instrument composed of 4 items per factor (8 items total). Convergent and discriminant validity evidence is also provided.

## 1. Introduction

In recent decades, the acceptance and inclusion of sexual minorities has improved. However, discrimination persists throughout society. Stigmatizing messages, attitudes, and behaviors are pervasive. This stigma not only emanates from the sexual majority but can also be internalized by the sexual minority, leading to difficulties for them and their relationships (e.g., family, friends, partners, etc.; Feinstein et al., 2012; Herek et al., 2009; Preston et al., 2007; Szymanski & Gupta, 2009; Szymanski & Ikizler, 2013; Szymanski & Mikorski, 2016; Szymanski & Sung, 2010). To better understand the impact of internalized sexual stigma on sexual minorities and their experiences and interactions with others, a measure of internalized sexual stigma for lesbian, gay, and bisexual (LGB) individuals is needed. To date, such a measure has not been developed. Therefore, the purpose of the present study is to develop and validate a measure of internalized sexual stigma for use with LGB individuals ages 18 and older.

Historically, sexual minorities have experienced not only discrimination but persecution. For instance, the legal system was used as a means of suppression. In the 1530s, homosexuality or anal intercourse could result in the death penalty. However, the death penalty was later deemed excessive, so sentencing was reduced to 10 years to life imprisonment. Clear boundaries were drawn between acceptable sexual behavior and deviant sexual behavior, i.e., conjugational heterosexuality and non-conjugational homosexuality, respectively. This hard legal stance on homosexuality led many to view it as a sin or mental illness (Weeks, 1999). Unfortunately, the American Medico-Psychological Association (circa 1892–1920), which later became the American Psychiatric Association (APA) in 1921, viewed homosexuality as a mental illness in their early publications. The Statistical Manual for the Use of Institutions for the Insane (American Medico-Psychological Association, 1918) classified homosexuality as a psychopathic personality with pathological sexuality. The subsequent Diagnostic and Statistical Manual (DSM-I; American Psychiatric Association, 1952) viewed homosexuality as a personality disorder and classified it as a sexual deviation within the sociopathic personality disturbance category. It was not until the seventh printing of the second edition of the DSM (DSM-II; American Psychiatric Association, 1968) that this diagnosis was removed.

This historical foundation laid the groundwork for sexual stigma towards sexual minorities. The term sexual stigma refers to shared societal beliefs that result in the lesbian, gay, bisexual, transgender, and queer (LGBTQ+) community being denigrated, discredited, and invalidated (Lingiardi et al., 2012). Sexual stigma also refers to the social processes of devaluation, negative attitudes, and stereotyping towards sexual minorities (Logie & Earnshaw, 2015). Although attitudes towards sexual orientation have evolved to increased acceptance of sexual minorities since the 19th century, sexual minorities are still victimized by discrimination and hostility (Herek et al., 2009). The LGBTQ+ community experiences widespread stigma and discrimination, which can be deleterious to individual well-being (Logie & Earnshaw, 2015). The pervasiveness of stigma can lead to internalized sexual stigma.

Internalized sexual stigma (ISS) is often referred to as internalized homonegativity, homophobia, or heterosexism (IH). These terms collectively encompass psychological and emotional responses by LGBTQ+ individuals who have adopted and internalized society’s negative beliefs, attitudes, and assumptions of homosexuality (Herek et al., 2009; Shidlo, 1994). Thus, sexual minorities can exhibit sexual stigma towards themselves as well as others (Herek et al., 2009). For instance, IH is characterized by shame and horizontal oppression, in which an LGB individual directs homonegativity towards another LGB individual (Szymanski & Chung, 2001).

The term sexual stigma can further be divided into two separate components: enacted stigma and perceived stigma. Enacted stigma is the overt experiences of discrimination, such as physical, verbal, and sexual violence (Logie & Earnshaw, 2015). Perceived stigma refers to an individual’s awareness of negative attitudes as well as treatment towards an individual’s group (Logie & Earnshaw, 2015). Previous studies have focused more on enacted stigma and demonstrated that it is associated with increased physical symptoms, negative mood, and exposure to stress in the same population (Logie & Earnshaw, 2015). Perceived stigma has been associated with mental health issues such as substance use, suicidal ideation, and emotional and psychological distress among lesbian women (McCabe et al., 2009; Shearer et al., 2016; Szymanski & Mikorski, 2016).

IH is of importance due to its psychological impact on the LGB community (Shidlo, 1994). Negative psychological effects include low self-esteem, depression, shame, self-hatred, and feelings of inadequacy and rejection (Weber-Gilmore et al., 2011). IH has also been found to be associated with somatic symptoms, wanting to pass as heterosexual, low self-stability, low social support, and low satisfaction with social support (Szymanski & Chung, 2001). Given the negative effects associated with IH, it is vital to have a valid measure of IH for LGB individuals. To date, at least six popular self-administered IH or ISS measures have been used to examine outcomes.

The first measure of IH is the Nungesser Homosexual Attitudes Inventory for gay men (NHAI; Newcomb & Mustanski, 2010; Nungesser, 1983; Szymanski et al., 2008). The original development of the NHAI was based on an initial pool of 84 true/false items measuring three constructs: (1) attitudes towards one’s homosexuality (self; 10 items), (2) attitudes towards other homosexuals (other; 12 items), and (3) attitudes towards the disclosure of one’s own homosexual identity (disclosure; 12 items). The items were completed by 50 gay men recruited from “public cruising areas” (p. 72) and a university gay activist meeting. The average age of the participants was 25; the *SD* was not reported. The initial pool was reduced to 34 items using the index of discrimination (*D*) with coefficient alphas of 0.94 for self, 0.88 for other, and 0.91 for disclosure. The 34 items were subsequently converted to a self-administered questionnaire with Likert-type items. The number of categories and corresponding labels were not provided. The 34 items were completed by 50 gay men from a gay community group and university faculty, from which an overall coefficient alpha of 0.95 was computed for the 34 items of the NHAI.

Shidlo (1994) was the first to review the self-administered NHAI content, suggest revisions, and report basic psychometric properties. Based on the content analysis, Shidlo proposed a 36-item revised NHAI (RNHAI) with modernized and clarified item wording for 30 of the original NHAI items and the addition of 6 items to the self construct. The items were completed by 62 gay and bisexual men aged 18–68 (*M* = 32.27, *SD* = 9.59) recruited from a New York City gay community center. Shidlo also reported an overall coefficient alpha of 0.90 for the RNHAI; coefficient alpha for each factor was not reported. As an index of discrimination, the analysis also included item-total correlations for each of the items, which ranged from 0–0.67. The RNHAI was related to AIDS-Related Internalized Homonegativity (r = 0.68), psychological distress (*r* = 0.43), self-esteem (*r* = −0.56), and social support (*r* = −0.25).

Rosario et al. (2001) modified the NHAI to a self-administered version that measures attitudes towards homosexuality. The measure was designed to accommodate bisexuals and included 3 items from the authors and 30 from the original NHAI (33 items) that were modified by “simplifying the language, making it more informal, and generalizing the item content to include females” (p. 143). The items were also rescaled from true/false to Likert-type with responses ranging from *disagree strongly* (1) to *agree strongly* (4), where higher scores indicated more positive attitudes towards homosexuality. The items were completed by 80 males and 76 females *n* = 156) aged 14–21 (*M* = 18.3, *SD* = 1.65) from New York City. Results indicated two factors (dimensions): attitudes towards homosexuality (*α* = 0.85) and comfort with homosexuality (*α* = 0.90); the factor extraction and rotation methods were not reported. For gay and bisexual males, the NHAI was related to self-esteem (*r* = 0.41), anxiety (*r* = −0.28), and depression (*r* = −0.41). For lesbian and bisexual females, the NHAI was related to depression (*r* = −0.24). In addition, the overall attitude towards homosexuality was related to social desirability (*r* = 0.22). Notably, the researchers did not specify which items comprise the factors, and the factor analysis was not the primary focus of the study.

A second popular measure is the 9-item Internalized Homophobia (IHP) Scale, created by Martin and Dean (1988). The scale was based on items from the diagnostic criteria for ego-dystonic homosexuality that focused on respondents’ attitudes about their sexual orientation (DSM-III; American Psychiatric Association, 1980). In addition, the scale was administered through face-to-face interviews with Likert-type response options from *never* (1) to *often* (4). Items were completed by 741 gay men aged 21–76 (*M* = 38, *SD* = 8.4) from New York City urban communities. Meyer (1995) reported an overall coefficient alpha of 0.79, and the IHP appeared to correlate with or predict distress variables as expected.

An early pilot study investigated predictors of high-risk sexual behaviors and aspects of psychological functioning that included the IHP (Herek & Glunt, 1995). The 9-item IHP was adapted as a self-administered questionnaire with response options of *never* (1) to *often* (4). The sample consisted of 65 gay and 29 bisexual males, aged 17–70 (*M* = 32; *SD* not reported) from a metropolitan area in Sacramento, CA. The authors reported a coefficient alpha of 0.83 for the IHP. The IHP was related to depression (+), self-esteem (-), collective self-esteem (-), community consciousness (-), importance attached to community involvement (-), disclosure or outness to heterosexual friends (-), and dissatisfaction with local gay/bisexual community (+). The specific correlations for each of these variables were not reported; however, it was indicated that the absolute value of each correlation was at least 0.30 (|*r*| ≥ 0.30).

Another early study investigated the correlates of IH using the IHP (Herek et al., 1998). The 9-item IHP was adapted to a self-administered questionnaire with response options changed to *disagree strongly* (1) to *agree strongly* (5). The sample consisted of 60 gay males, 66 lesbian females, and 21 bisexuals ages 16–68 (*M* = 33; *SD* not reported) from a street fair in Sacramento, CA. Herek et al. (1998) reported IHP coefficient alphas of 0.83 and 0.71 for males and females, respectively. However, bisexual individuals were excluded from further analyses because of their small sample size, and they scored significantly higher than gay/lesbian individuals on the IHP. For gay/lesbian individuals, IHP was significantly related to self-disclosure (*r* = −0.30 to −0.34) and sense of connection to the sexual minority community (i.e., lesbian & gay) (*r* = −0.38 to −0.47). For gay males only, IHP was related to depressive symptoms (*r* = 0.27), demoralization (*r* = 0.40), and self-esteem (*r* = −0.45).

A shorter version of the IHP was first used by Herek et al. (2009). The 5-item Revised Internalized Homophobia Scale (IHP-R) is a smaller subset of the 9-item IHP that was obtained through “a series of factor- and item analyses” (p. 36) and is more appropriate for lesbians and bisexuals. The IHP-R is a self-administered measure with response options that range from *disagree strongly* (1) to *agree strongly* (5). The sample consisted of 2259 LGB individuals (1170 females and 1089 males) from the greater Sacramento, CA area; age was not reported. Notably, the authors had to log transform the items because the *disagree strongly* (1) response was inflated, i.e., a floor effect. The authors reported coefficient alphas of 0.82 and 0.85 for the IHP-R and IHP, respectively, with correlations greater than 0.90 between the IHP-R and IHP for all sexual minority groups. The IHP-R was significantly positively related to depressive symptoms (*β* = 0.27) and negatively related to self-esteem (*β* = −0.27). In addition, the IHP-R was significantly negatively related to affect about their membership in the sexual minority community (i.e., lesbian, gay, & bisexual; *β* = −0.39) and outness to the world (i.e., heterosexual friends, acquaintances, etc.; *β* = −0.23).

A recent study investigated gender measurement invariance for the 5-item IHP-R (Huynh et al., 2020). In the study, gender invariance was compared between the 9-item IHP and 5-item IHP-R. Both scales were self-administered with response options that ranged from *strongly disagree* (1) to *strongly agree* (5). The sample consisted of 118 male and 163 female sexual minorities aged 19–76 (*M* = 33.04, *SD* = 10.53) recruited from online sites including professional listservs, Craigslist, Facebook, etc. It is unclear if bisexual individuals were included in the sample. A one-factor CFA was fit to each of the two scales, and the results indicated that the IHP-R had a better fit. Subsequently, the one-factor IHP-R was tested for gender invariance. Results indicated that the one-factor IHP-R met configural, metric, and scalar invariance. Coefficient alpha was not reported.

A third measure is the Internalized Homophobia Scale (IHS; Wagner et al., 1994), a 20-item self-administered measure adapted from the NHAI. The IHS consists of 9 items from the NHAI and 11 items written by the authors. Participants indicated their level of agreement on a 5-point scale; corresponding labels were not provided. The IHS was completed by Catholic gay males from the 1991 Dignity Biennial National Convention (*n* = 48), the New York Dignity organization (*n* = 53), and a New York City community sample without membership in gay church organizations (*n* = 45). The average age of the participants was 40; the *SD* was not reported. Wagner et al. reported an overall coefficient alpha of 0.92 for the IHS. For the Dignity Convention and NY Dignity sample, IHS was significantly related to demoralization (*r* = 0.38, 0.49). In addition, the IHS for the overall sample was significantly related to political views (*r* = −0.35), religious outlook (*r* = −0.51), and integration into the gay community (*r* = −0.54).

A fourth measure is the Internalized Homonegativity Inventory (IHNI) that measures internal homonegativity in gay men (Mayfield, 2001). The author developed an initial pool of 40 items, which were then reviewed and revised by members of a counseling psychology program: 4 gay male doctoral students, 1 bisexual female doctoral student, and 3 non-gay male professors. This resulted in a 42-item self-administered measure with response options of *strongly disagree* (1) to *strongly agree* (6). The measure was completed by 241 gay men aged 18–66 (*M* = 33.9, *SD* = 9.6) from the US recruited from local gay bars, adult bookstores, churches with primarily sexual minority membership, gay choirs, and the internet. An exploratory factor analysis using principal axis factoring with a promax rotation indicated three factors: 11 items for personal homonegativity, 7 items for gay affirmation, and 5 items for morality of homosexuality. The overall coefficient alpha was 0.91 and greater than 0.70 for each factor, with factor correlations ranging from 0.40–0.51. In addition, the factors were related to the NHAI (*r* = 0.53 to 0.82), extroversion (*r* = −0.14 to −0.24), emotional stability (neuroticism) (*r* = 0.15 to 0.29), and gay identity (*r* = −0.39 to −0.65). Gay affirmation was not related to emotional stability, and none of the factors were related to social desirability.

The fifth measure is the Lesbian Internalized Homophobia Scale (LIHS) that measures IH in lesbian women (Szymanski & Chung, 2001). Based on the literature, published scales related to the topic, and the author’s expertise, an initial pool of 73 self-administered items was developed, reflecting 5 dimensions with response options ranging from *strongly disagree* (1) to *strongly agree* (7). Prior to administering the items to participants, some items were removed based on a lack of consensus among 5 judges familiar with the lesbian literature; the number of removed items was not reported. The remaining items were completed by 303 lesbian females aged 18–65 (*M* = 34.64; *SD* not reported), mainly from the US, recruited from listservs, friendship networks, and snowball sampling. Of note is that the sample included participants who were bisexual but primarily lesbian (18.2%), bisexual but primarily heterosexual (7%), heterosexual (3.6%), and other (0.7%). Based on item-total correlations less than 0.35, additional items were removed, resulting in a 52-item measure. The item-total correlations for the 52-item LIHS ranged from 0.38–0.76. Szymanski reported an overall coefficient alpha of 0.94 for the LIHS with the following 5 factors: connection with the lesbian community (*α* = 0.87), public identification as a lesbian (*α* = 0.92), personal feelings about being a lesbian (*α* = 0.79), moral and religious attitudes toward lesbians (*α* = 0.74), and attitudes toward other lesbians (*α* = 0.77). Factor correlations ranged from 0.37–0.57. In addition, the LIHS and its factors were significantly related to self-esteem (*r* = −0.20 to −0.31) and loneliness (*r* = 0.14 to 0.41). Moral and religious attitudes toward lesbians and attitudes toward other lesbians were not related to self-esteem.

Lastly, the 17-item Measure for Internalized Sexual Stigma for Lesbians and Gay Men (MISS-LG) was the first measure developed for both lesbian females and gay males (Lingiardi et al., 2012). An initial pool of 46 female and 47 male items was developed based on clinical interviews with 15 gay men, 15 lesbian females, and a focus group of 20 field experts (i.e., psychologists, psychotherapists, and nonacademic LG community members). Subsequently, three clinical and developmental psychology researchers defined the initial pool of items as encompassing 3 factors (dimensions): identity, social discomfort, and sexuality. The item pool was reduced to 22 items, with response options ranging from *I disagree* (1) to *I agree* (5). The 22 items were completed by 67 lesbian females and 93 gay males aged 25–40 from Rome, Italy. A factor analysis indicated 3 factors: identity (5 items), social discomfort (7 items), and sexuality (5 items); however, the factor extraction and rotation methods used were not reported. The 17 items retained were subsequently completed by 335 lesbian females and 400 gay males, with an average age of 28.96 (SD = 7.19), from Italian websites and LGBTQ+ organizations. A three-factor confirmatory factor analysis with item parceling was fit to females and males. For lesbian females, the model fit was *χ*^2^(24, *N* = 335) = 98.21, RMSEA = 0.05, AGFI = 0.90; for gay males, the model fit was *χ*^2^(24, *N* = 400) = 70.70, RMSEA = 0.04, AGFI = 0.91. Factor correlations ranged from 0.40–0.56, and coefficient alphas ranged from 0.77–0.80. In addition, the MISS-LG factors were significantly related to IHP (*r* = 0.32 to 0.52), self-disclosure to family (*r* = −0.24 to −0.48), self-disclosure to friends (*r* = −0.24 to −0.54), social anxiety (*r* = 0.15 to 0.48), and well-being (*r* = −0.19 to −0.45). Identity was not related to self-disclosure to family, self-disclosure to friends, or well-being.

### 1.1. Summary and Limitations of Presented Measures

Five issues can be identified from existing research. First, except for the MISS-LG, all the measures were initially developed for either gay males or lesbian females. Second, there is a lack of information on the factor structure for most measures. Exploratory factor analysis (EFA) was performed for only four measures, the NHAI, IHP-R, IHNI, and MISS-LG; furthermore, EFA details were only provided for the IHNI. Confirmatory factor analysis was only performed for the IHP-R and MISS-LG. Third, none of the measures were initially developed to include bisexual individuals. Although the NHAI and IPH/-R were later adapted to include bisexual individuals, bisexual individuals were separated into males and females in the corresponding analyses. Additionally, the adaptation included adjustments to the wording of some items. For example, the IHP has separate items for lesbian and gay individuals, i.e., two sets of items. Fourth, only the IHP-R has been tested for measurement invariance between sex (i.e., males and females), and it is unclear if bisexual individuals were included in the sample. Finally, except for the LIHS and MISS-LG, all measures were initially developed with local convenience samples. The MISS-LG was created with an Italian sample, whereas the remaining measures utilized U.S. samples. This means that none of the measures have been tested for measurement invariance for sexual orientation (i.e., LGB individuals). Therefore, it is unclear how appropriate these measures are for LGB individuals. Although bisexual individuals can experience unique discrimination experiences as compared to lesbian females and gay males, it is important to consider a measure that includes internal stigma for bisexual individuals (Brewster et al., 2013). Huynh et al. (2020) have suggested that the literature would benefit from such a measure. As such, the present study aims to develop and validate a measure of ISS for LGB individuals.

### 1.2. Objective

Based on the summary above, the objective of this study is to develop and validate an alternative measure of ISS with a clear factor structure appropriate for lesbian, gay, and bisexual individuals. To this end, two studies were conducted. In Study 1, an initial item pool was developed based on a literature review and interviews with LGB individuals. The initial item pool was explored to extract a simple factor structure. In Study 2, the factor structure from Study 1 was tested for confirmation. Additionally, the psychometric properties of the items from the confirmed factor structure were established. Lastly, measurement invariance between the LGB groups was tested to investigate whether the confirmed factor structure of the items operates equivalently for lesbian, gay, and bisexual individuals.

## 2. Study 1: Methods

Study 1 focused on developing an initial item pool and exploring its factor structure. An EFA and corresponding statistics were computed.

### 2.1. Study 1: Procedure

All participants were self-identified LBG individuals ages 18 years or older. Interview participants signed informed consent forms and were recruited from a local university, various locations around the metropolitan and surrounding rural areas, and through snowball sampling. Local university participants were recruited from the university’s Participation Pool via Sona Systems (https://www.sona-systems.com/; accessed on 1 July 2025). All other participants were recruited from Amazon Mechanical Turk (MTurk). Participants read a description of the study and were informed that they would be asked to share their perspective on sexual stigma; subsequently, they filled out an anonymous online survey. After completing the study, university student participants received course credit as an incentive, and all others received a monetary incentive. The first author’s Human Subjects Review Committee (HSRC) approved the research.

### 2.2. Study 1: Participants

For the initial item pool development, a total of 50 self-identified LGB individuals were interviewed, aged 19–64 (*L*: *N* = 24, *M* = 29.08, *SD* = 5.50; *G*: *N* = 13, *M* = 33.31, *SD* = 9.67; *B*: *N* = 13, *M* = 29.69, *SD* = 11.91).

These items were then completed by qualified participants (87 lesbian females, 63 gay males, 73 bisexual females, and 101 bisexual males; *N* = 327) ages 18–62 (*M* = 29.15, *SD* = 6.45). Respondents were White (112; 34.6%), Black/African American (22; 6.8%), Asian (97; 29.9%), Hispanic (72; 22.2%), Other (12; 3.7%), or did not report ethnicity (9; 2.8%).

### 2.3. Study 1: Item Development and Exploratory Stage

An initial item pool was generated for the ISS measure based on a literature review and interviews with LGB individuals. All sessions were transcribed and analyzed for themes and patterns to develop items.

### 2.4. Study 1: Measures

The initial ISS item pool consisted of 24 items. Sample items were “I wish I could change my sexual orientation” and “I am uncertain how others will react to my sexual orientation.” Participants rated each item on a Likert-type scale ranging from *strongly disagree* (1) to *strongly agree* (6).

### 2.5. Study 1: Data Checking

There were no missing data. In terms of normality, all variables had acceptable skewness and kurtosis for factor analysis, i.e., skewness between −2 and 2 and kurtosis between −7 and 7 (Curran et al., 1996).

## 3. Study 1: Results

### Study 1: Exploratory Factor Analysis (EFA)

An EFA was used to determine the factor structure of the initial 24-item pool. Factors were extracted using principal axis factoring and then rotated with a Promax rotation, which allows rotated factors to correlate. The primary consideration in all EFAs was an interpretable factor structure with clear factor loadings (i.e., simple structure; Tabachnick & Fidell, 2007).

Although the scree plot for the initial item pool suggested three factors, two factors were extracted and rotated, as this approach led to a more interpretable factor structure. Of the 24 items, 10 items were removed due to loadings below 0.30 (Gorsuch, 1983), cross-loadings, or not conceptually fitting a factor. The final factor structure of the remaining 14 items is presented in Table 1, and the corresponding coefficient alpha estimates with normal theory bootstrap confidence intervals (CIs; Padilla et al., 2012) in Table 2. A clear factor structure was achieved, and all factors had strong internal consistency.

Based on the item loadings, two interpretable factors emerged. The first factor is internal conflict with corresponding items pointing to an LGB individual’s distress with their sexual orientation. Distress interferes with an LGB individual’s ability to fit in with a stigmatizing society and interact with other individuals. The second factor is disclosure conflict with corresponding items pointing to an LGB individual’s distress based on their expectation of rejection. The two factors have a strong correlation (*r* = 0.70). The correlation indicates that LGB individuals perceive and are aware of society’s negative attitudes towards the LGB community. For example, if an LGB individual fears being judged for their sexual orientation, they may also feel anger towards their sexual orientation. As such, the two factors collectively measure the construct of internalized sexual stigma in the Perceived Internalized Sexual Stigma Measure (PrISSM).

## 4. Study 1: Discussion

Informed by the literature, interviews, and an EFA, an initial 14-item Perceived Internalized Sexual Stigma Measure (PrISSM) was developed, consisting of two factors: (a) internal conflict and (b) disclosure conflict. With respect to the measure, LGB individuals perceive or are aware of society’s stigma towards the LGB community, which is reflected in the two PrISSM factors and their relationship. As such, LGB individuals expect to be rejected by a stigmatizing society, which is reflected in the disclosure conflict factor. In addition, an LGB individual’s distress with fitting in with a stigmatizing society creates an internal conflict reflected by the internal conflict factor. Lastly, the 14-item PrISSM demonstrated good initial psychometric properties.

## 5. Study 2: Methods

Study 2 focused on confirming the 14-item PrISSM factor structure identified in Study 1 as well as establishing convergent and discriminant validity. A Bayesian confirmatory factor analysis (BCFA) and corresponding supporting statistics were computed.

### 5.1. Study 2: Procedure

Participants in Study 2 were a separate and independent sample from those in Study 1. All participants were self-identified LGB individuals aged 18 years and older recruited from Amazon Mechanical Turk (MTurk). Participants read a description of the study and were informed that they would be completing an online survey about their perspective on sexual stigma. After completing the survey, all participants received a monetary incentive. The first author’s HSRC approved the research.

### 5.2. Study 2: Participants

A battery of measures was administered to qualified participants (118 lesbian females, 124 gay males, 132 bisexual females, and 132 bisexual males; *N* = 506) ages 18–82 (*M* = 35.08, *SD* = 13.64). Respondents were White (268; 53.0%), Black/African American (42; 8.3%), Asian (91; 18.0%), Hispanic (85; 16.8%), Other (18; 3.5%), or did not report ethnicity (2; 0.4%).

### 5.3. Study 2: Measures

Participants were given the 14-item PrISSM from Study 1, the 9-item IHP, the 5-item IHP-R, and the 30-item Big Five Inventory-2 Short form (BFI-2-S; Soto & John, 2017). As measures of internalized homophobia, the IHP/-R was administered to provide evidence of convergent validity. The IHP/-R has acceptable internal consistency (*α* = 0.83 for men, *α* = 0.71 for women) and correlates as expected with relevant measures (Herek et al., 1998; Herek & Glunt, 1995). IHP/-R items are measured with a Likert scale ranging from *strongly disagree* (1) to *strongly agree* (5). For female participants, the following item words were changed for the IHP/-R: “gay” to “lesbian,” and “men” to “women.” For example, “I wish I weren’t gay/bisexual” was changed to “I wish I weren’t lesbian/bisexual.”

The BFI-2-S is a distinct instrument that measures personality via the following 5 constructs with corresponding average coefficient alphas: extraversion (*α* = 0.78), agreeableness (*α* = 0.75), conscientiousness (*α* = 0.76), open-mindedness (*α* = 0.76), and negative emotionality (*α* = 0.83). Each BFI-2-S construct consists of 6 items measured with a Likert scale ranging from *disagree strongly* (1) to *agree strongly* (5).

The BFI-2-S was used for three reasons. First, as a shorter version of the BFI, the BFI-2-S reduces participant burden without reducing its psychometric properties. Second, evidence indicates that the BFI constructs are stable over time (Atherton et al., 2022). This helps reduce the chance that any relationship, or lack of relationship, with the BFI constructs is based on episodic situations or circumstances. Finally, the BFI will provide evidence of convergent and discriminant validity.

In terms of validity, different relationship patterns between the BFI-2-S and PrISSM were investigated. Regarding convergent validity, agreeableness and open-mindedness were expected to be strongly related to internal conflict, while negative emotionality was expected to be strongly related to disclosure conflict. Agreeableness entails being compassionate, caring, and respectful of others and oneself. As such, more agreeable individuals would be more accepting of their sexual orientation (i.e., have less internal conflict). Open-mindedness reflects an interest in the arts, creative thinking, and generating new ideas. Hence, more open-minded individuals would be less conflicted about their sexual orientation. Negative emotionality is characterized by being easily upset and having difficulty handling stress. From this perspective, individuals with more negative emotionality would tend to be more anxious about others’ reactions to their sexual orientation (i.e., more disclosure conflict).

Regarding discriminant validity, weak or no relationships were expected between the BFI-2-S and PrISSM constructs/factors. First, based on the previous descriptions of agreeableness, open-mindedness, and negative emotionality, it was expected that more agreeable individuals would be less inclined to believe that others are judging or discriminating against them based on sexual orientation (less disclosure conflict). More open-minded individuals would also be less likely to be boxed into established norms (i.e., less disclosure conflict). On the other hand, an individual who is internally conflicted does not necessarily translate into one who is easily upset and/or unable to handle stress (i.e., negative emotionality). Conscientious individuals are considered reliable, persistent on task, organized, and neat. As such, conscientious individuals who are task-oriented and focused on task completion might be less likely to ruminate about their sexual orientation (i.e., less internal conflict) and less concerned with social approval (i.e., less disclosure conflict). Extraversion reflects individuals who are outgoing and sociable, often apt to take on leadership roles. As such, extraverted individuals are more concerned with connecting with others and less concerned about others’ judgments or letting others set the tone or path (i.e., less disclosure conflict). Lastly, given that extraversion deals with how an individual interacts with others socially, this does not necessarily translate into internal thoughts and ideas (i.e., internal conflict). It was expected that the relationships here would be weak or nonexistent.

### 5.4. Study 2: Data Checking

Listwise deletion was used to address the small amount of missing data, i.e., a loss of two participants. Consequently, the sample sizes will slightly differ for each of the analyses. In terms of normality, all variables had acceptable skewness and kurtosis for factor analysis, i.e., skewness between −2 and 2 and kurtosis between −7 and 7 (Curran et al., 1996).

## 6. Study 2: Results

### 6.1. Study 2: PrISSM Bayesian Confirmatory Factor Analysis (BCFA)

The 14-item PrISSM factor structure from Study 1 was tested with a Bayesian confirmatory factor analysis (BCFA), and corresponding supporting statistics were also estimated. BCFA model fit was assessed using the following criteria: the Bayesian comparative fit index (BCFI) ≥ 0.95, Bayesian gamma hat (BGH) ≥ 0.95, and posterior predictive *p*-value (PPP) close to 0.5 (Hu & Bentler, 1999; Garnier-Villarreal & Jorgensen, 2020). In traditional structural equation modeling, the chi-squared of model fit has two drawbacks: it is (1) sensitive to sample size and (2) allows for model overfitting. As such, fit indices are used to supplement model fit (e.g., CFI). The PPP has the identical drawback as the chi-squared test for model fit, so Bayesian fit indices are used to supplement the model fit (e.g., BCFI and BGH). Model comparisons were assessed using the leave-one-out cross-validation (LOO-CV), where lower values indicate a better-fitting model (Garnier-Villarreal & Jorgensen, 2020). In addition, the LOO-CV has a corresponding expected log-pointwise-predictive-density (ELPD) and standard error (SE), which can form a RATIO of ELPD over SE to compare models. A RATIO ≥ 2 provides evidence of differences in models. All relevant Bayesian estimates will have corresponding highest probability density intervals that will be referred to as highest density intervals (HDIs) for conciseness. All BCFAs were estimated using three chains of 6000 posterior samples each, 1000 to establish a stationary distribution (i.e., burn-in samples) and 5000 post-burn-in for inference. Noninformative (or diffused) priors were used for all model parameters.

The PrISSM BCFA fit and modification indices (MIs) for each step of the analyses are in Table 3. The initial 14-item BCFA in Step 1 indicated that the two-factor structure had a decent fit. Even so, it is common for initial structural equation models to be refined (Kline, 2023; Loehlin & Beaujean, 2017). To refine the factor structure, items with MIs ≥ 9 were removed in successive steps. Model refinement ended at the final BCFA in Table 3.

In Table 3, items were removed due to redundancy and/or tapping on aspects of both factors. Steps 1, 3, and 4 consisted of redundant items. For example, the items in step 4 are similar to one another. However, item 12 was removed because it was specific to disclosure conflict with the family, and the items in the disclosure conflict factor are reflective of disclosure conflict with society outside of the family. On the other hand, steps 2, 5, and 6 consisted of items that tapped into aspects of both factors. For example, item 6 in step 2 taps into internal and disclosure conflict, as being internally conflicted does not allow someone to fit in and thus disclose their sexual orientation. Consequently, item 6 was removed.

An 8-item one-factor model was estimated and had the following fit: PPP = 0.000, BCFI = 0.0.736 [0.733, 0.740], BGH = 0.760 [0.757, 0.762], and LOO-CV = 14,074.540; numbers in brackets are 90% HDIs. The final 8-item two-factor structure was compared to the 8-item one-factor structure, in which the LOO-CV ELPD difference RATIO indicated that the two- and one-factor BCFAs fit the data differently, with ELPD = 312.65 and SE = 35.41, with RATIO = 8.829, i.e., the two-factor model has a better fit.

The 8-item PrISSM standardized factor loadings are presented in Table 4, along with corresponding coefficient alpha estimates and normal theory bootstrap confidence intervals (CIs; Padilla et al., 2012), as shown in Table 5. Factor loadings were all *excellent*, ranging from 0.73 to 0.90 (Comrey & Lee, 1992). The PrISSM and IHP/-R had good internal consistency with coefficient alphas significantly greater than 0.70 (Nunnally & Bernstein, 1994). However, the BFI-2-S extraversion coefficient alpha fell short of 0.70.

### 6.2. Study 2: Other PrISSM Validity Evidence

Correlations between the IHP/-R and BFI-2-S were computed to assess the validity of the PrISSM and are presented in Table 6. Note that each factor in Table 6 is the sum of the corresponding items. The PrISSM internal and disclosure conflict factors correlated strongly (*r* = 0.48, *p* < 0.001), indicating that they are dimensions of internalized sexual stigma, as shown in the results of Study 1. In addition, the IHP/-R were strongly correlated with the PrISSM and its corresponding factors. Given that internal conflict was strongly correlated with the IHP/-R (*r* = 0.89, 0.89, *p* < 0.001, *p* < 0.001, repectively), its correlation pattern with the BFI-2-S was virtually identical to that of the IHP/-R. The correlation patterns of the BFI-2-S with the PrISSM were supported. For convergent validity, agreeableness and open-mindedness were strongly correlated with internal conflict. Additionally, negative emotionality was strongly correlated with disclosure conflict. For discriminant validity, the remaining correlations were small (Cohen, 1988) even though most of them were significant, which in this case was a function of the sample size (N = 504).

### 6.3. Study 2: PrISSM Bayesian Measurement Invariance

Three measurement invariance procedures with three models each were conducted for the final 8-item PrISSM. Results of the measurement invariance analyses are presented in Table 7. First, measurement invariance was investigated between lesbian females and gay males. Equality of the unstandardized factor structure across the groups was examined with a configural invariance model. In the configural model, the 8-item two-factor BCFA was simultaneously estimated for each corresponding group. According to the results, the configural model has a good fit. As such, the number of factors and factor loading patterns are equivalent across lesbian female and gay male groups. Therefore, a subsequent metric model was estimated to examine a difference in model fit.

Equality of the unstandardized item factor loadings across the groups was then examined with a metric (weak) invariance model. All loadings were set to be equal across the groups. The metric model had a good fit and did not significantly differ in fit from the configural model (RATIO = 1.71). As such, the items relate to the factors equivalently across lesbian females and gay males. Therefore, a subsequent scalar model was estimated to examine a difference in model fit.

Equality of the unstandardized item intercepts across the groups was then examined with a scalar (strong) invariance model. All loadings and all intercepts were set to be equal across the groups. The scalar model had a good fit and did not differ significantly in fit from the metric model (RATIO = 0.82). As such, the same item responses are expected at the same absolute trait level for lesbian females and gay males.

Second, measurement invariance was investigated between male and female bisexual individuals. According to the results, the configural model has a good fit. The metric model had a good fit and did not significantly differ in fit from the configural model (RATIO = 1.89). The scalar model also had a good fit and did not differ substantially in fit from the metric model (RATIO = 1.03).

Given that measurement invariance held between lesbian females and gay males separately from male and female bisexuals, these groups were combined. Specifically, lesbian females and gay males were combined into lesbian-gay (LG) individuals. In addition, male and female bisexual individuals were combined into bisexual (B) individuals.

Third, measurement invariance was tested for LG and B individuals. The results indicate that the configural model has a good fit. The metric model also had a good fit and did not significantly differ in fit from the configural model (RATIO = 0.91). The scalar model also had a good fit and did not differ substantially in fit from the metric model (RATIO = 0.85).

In summary, the analysis showed that the 8-item PrISSM is invariant for LGB individuals. In other words, the number of factors, factor loading patterns, and intercept magnitude patterns are equivalent across LGB individuals. The same item responses are expected at the same absolute trait level for LGB individuals. This indicates that the 8-item PrISSM structure works equivalently for LGB individuals, i.e., measures internalized sexual stigma equivalently for LGB individuals.

## 7. Discussion

It is important to consider internalized sexual stigma (ISS) as it is a contributor to psychological distress in LGB individuals (Herek et al., 2009; Szymanski & Gupta, 2009; Szymanski & Sung, 2010). Although existing measures have contributed to understanding internalized homophobia and its impact on sexual minorities, most of these measures only focus on one sex (i.e., male or female) or at most two sexual orientations (e.g., lesbians and bisexuals). Additionally, the factor structure information for most of these measures is lacking. Along this same line, it is unclear whether the factor structures of these measures are equivalent across LGB individuals, i.e., invariant across LGB individuals. The purpose of this study was to develop an alternative ISS measure appropriate for LGB individuals, the Perceived Internalized Sexual Stigma Measure (PrISSM). Findings indicate that the PrISSM and its factors (dimensions) have sound psychometric properties.

An EFA and CFA revealed that the PrISSM was composed of two factors: internal conflict and disclosure conflict. In addition, the correlation between the two PrISSM factors indicates that LGB individuals perceive and are aware of society’s negative attitudes towards the LGB community. This perception and awareness point to LGB individuals expecting to be rejected (disclosure conflict) from a stigmatizing society, from which they struggle to fit in (internal conflict). These two factors are similar to 2 of the 3 factors initially proposed by for the NHAI (Nungesser, 1983): attitudes towards one’s homosexuality (*self*) and attitudes towards the disclosure of one’s homosexuality (*other*). However, unlike the NHAI, which generalizes to only gay men, the PrISSM generalizes to LGB individuals. It appears that internalizing sexual stigma from a heterosexual-majority society creates an internal conflict for LGB individuals, including the conflict of disclosing one’s sexual orientation to others.

The PrISSM also demonstrated convergent and discriminant validity. With respect to convergent validity, relationships of the PrISSM with the IHP/-R and BFI-2-S were presented. First, the PrISSM constructs were strongly related in the same direction to the IHP/-R. Given that the PrISSM internal conflict construct is more like the IHP/-R, it had a stronger relationship with the IHP/-R. Second, the PrISSM constructs had the expected relationships with the corresponding BFI-2-S constructs. In this respect, individuals who are respectful of others and self, or who are creative, tend to be less conflicted about their sexual orientation (i.e., less internal conflict). Additionally, individuals who are easily upset and have difficulty handling stress tend to be more anxious about others’ reactions to their sexual orientation (i.e., more disclosure conflict).

In terms of discriminant validity, the PrISSM demonstrated the expected small or no relationships with the corresponding BFI-2-S constructs. In terms of small relationships, individuals who are task-oriented and focused on task completion are not likely to ruminate about their sexual orientation. Additionally, individuals who are respectful of others and self, or who are creative, tend to be less likely to be boxed into established norms (i.e., less disclosure conflict). Furthermore, individuals who are reliable and organized, or who take on leadership roles, tend to be less likely to let others set the tone or path (i.e., less disclosure conflict). In terms of no relationships, an internally conflicted individual is not necessarily one who is unable to handle stress or interact socially with others. Collectively, these findings support the initial convergent and discriminant validity of the PrISSM.

Regarding measurement invariance, the PrISSM factor structure is appropriate for LGB individuals as it demonstrated configural, metric, and scalar invariance across these individuals. Meeting configural invariance indicates that the number of factors and factor loadings are equivalent across LGB individuals. Furthermore, meeting metric invariance allows for comparing the relationship between the PrISSM and other variables across LGB individuals. Lastly, meeting scalar invariance allows for the comparison of the PrISSM factor (latent variable) means or total factor scores across LGB individuals, as such differences are not influenced by LGB orientation.

There are benefits to the development of the PrISSM. First, having a multidimensional and psychometrically sound measure will facilitate a deeper understanding of the multidimensional nature of ISS across LGB individuals. Second, the PrISSM could be used in a clinical setting to assess ISS in LGB individuals, helpint to traget treatment, support, and personal goal setting. As noted in a recent article, self-esteem, self-worth, and a sense of self-efficacy are vital to overall health (Thimm-Kaiser et al., 2023). This can also extend to assessing ISS in organizations like schools and the workplace to help develop stigma-reducing initiatives and programs. Third, the PrISSM could be part of research investigating the complex interplay between sexual orientation, class, and social and economic factors. This is particularly important as gender non-conforming sexual minorities have been shown to have a lower socioeconomic status (Hernandez et al., 2024).

Three limitations are noted. First, test–retest reliability was not investigated. As such, it is unclear how the PrISSM will perform over a time interval. Even so, given that ISS takes years, if not decades, to develop, it is highly unlikely to change significantly over the time span of a research study, particularly in the absence of intervention. Second, the internal consistency was not ideal for some of the BFI-2-S constructs (e.g., extraversion). As such, the magnitude of the correlations of these constructs with other constructs may be impacted (see Table 6). The BFI-2-S was chosen to reduce participant burden. However, there may be some unique aspects of LGB individuals that impact the internal consistency of the BFI-2-S. For example, LGB individuals have likely been stigmatized their entire lives, and the BFI-2-S extraversion construct may not sufficiently capture the deeply rooted negative emotions associated with this stigmatization. Regardless, the correlation patterns of the BFI-2-S with the PrISSM are intuitively meaningful, including the correlations for extraversion and negative emotionality that are like those for the IHNI (Mayfield, 2001). Finally, although Amazon MTurk participants are commonly used in social sciences research, concerns have been raised about the generalizability of MTurk workers to the wider population. One study indicated that MTurk workers are similar to the wider population in terms of race and gender but not religion (Burnham et al., 2018).

## 8. Conclusions

In summary, the PrISSM is the first validated ISS measure designed explicitly for LGB individuals aged 18 years and older. Given its applicability to lesbian, gay, and bisexual individuals, it removes the need for differently worded, separate sets of items for people within this group. As an alternative measure of sexual stigma, the PrISSM can be used in conjunction with other IH measures. For example, for pretest–post-test studies, using an alternate measure can eliminate or reduce carryover (or learning) effects. Furthermore, as an 8-item measure, the brief nature of the PrISSM makes it ideal for studies that need to minimize participant burden. Given the findings and discussion, it is hoped that the PrISSM will contribute to advancing research on LGB individuals.

## Figures and Tables

**Table 1:** Perceived Internalized Sexual Stigma Measure (PrISSM) Final Factor Structure: Standardized Loadings.

Items	F1: Internal Conflict	F2: Disclosure Conflict
People that accept their sexual orientation frustrate me.	**0.83**	−0.02
I do not like interacting with others because of my sexual orientation	**0.81**	0.05
I am angry because of my sexual orientation	**0.81**	0.03
I have thought about committing suicide because of my sexual orientation	**0.80**	0.02
I wish I could change my sexual orientation	**0.79**	−0.01
I am unable to fit in with others because of my sexual orientation	**0.75**	0.10
I hope my sexual orientation would change	**0.71**	0.16
I fear that people will judge me for my sexual orientation	−0.01	**0.82**
My uncertainty of others’ possible reactions leads me to hide my sexual orientation	0.05	**0.80**
I am fearful of disapproval from others because of my sexual orientation	0.05	**0.78**
Facing discrimination because of my sexual orientation makes me anxious	−0.03	**0.77**
My family’s reaction to my sexual orientation worries me	0.06	**0.76**
I am uncertain how others will react to my sexual orientation	−0.01	**0.74**
The thought of coming out in my workplace gives me anxiety	0.07	**0.70**
Sum of Squared Loadings (SSL)	4.62	4.44
Proportion of Total Variance	0.33	0.32
Proportion Explained	0.51	0.49

N = 324 for the exploratory factor analysis. F1 & F2 correlation = 0.70. Bold numbers indicate strong loadings.

**Table 2:** Study 1 Internal Consistency Estimates.

Factor/Latent Variable	Items	Coefficient Alpha [95% CI]
PrISSM (F1, F2)	14	0.95 [0.94, 0.96]
Internal Conflict (F1)	7	0.94 [0.92, 0.95]
Disclosure Conflict (F2)	7	0.92 [0.90, 0.94]

N = 324 for all coefficient alphas. Confidence intervals are normal bootstrap based with 1000 bootstrap samples.

**Table 3:** Perceived Internalized Sexual Stigma Measure (PrISSM) Bayesian Confirmatory Factor Analysis Fit.

Steps	Modification Index
Step 1: PPP = 0.000, BCFI = 0.941 [0.939, 0.943], BGH = 0.915 [0.912, 0.918], LOO-CV = 22,490.91 Residual (error) correlation between items 5 & 7; dropped item 5 item 5: I wish I could change my sexual orientation. item 7: I hope my sexual orientation would change.	90.15 [83.88, 96.26]
Step 2: PPP = 0.000, BCFI = 0.954 [0.952, 0.957], BGH = 0.936 [0.933, 0.940], LOO-CV = 21,070.87 Cross-load item 6 on Factor 2; dropped item 6 item 6: I am unable to fit in with others because of my sexual orientation. Factor 2: Disclosure Conflict	46.18 [25.05, 66.84]
Step 3: PPP = 0.00, BCFI = 0.970 [0.967, 0.973], BGH = 0.959 [0.956, 0.962], LOO-CV = 19,615.61 Residual (error) correlation between items 11 & 9; dropped item 11 item 11: Facing discrimination because of my sexual orientation makes me anxious. item 9: My uncertainty of others’ possible reactions leads me to hide my sexual orientation	24.16 [14.17, 33.20]
Step 4: PPP = 0.000, BCFI = 0.979 [0.976, 0.982], BGH = 0.972 [0.968, 0.975], LOO-CV = 18,048.49 Residual (error) correlation between items 12 & 8; dropped item 12 item 12: My family’s reaction to my sexual orientation worries me. item 8: I fear that people will judge me for my sexual orientation.	21.98 [12.39, 30.56]
Step 5: PPP = 0.000, BCFI = 0.986 [0.983, 0.989], BGH = 0.981 [0.977, 0.985], LOO-CV = 16,412 Cross-load item 2 on factor 2; dropped item 2 item 2: I do not like interacting with others because of my sexual orientation. Factor 2: Disclosure Conflict	18.01 [6.86, 29.12]
Step 6: PPP = 0.012, BCFI = 0.991 [0.987, 0.994], BGH = 0.988 [0.984, 0.992], LOO-CV = 14,954.73 Cross-load item 10 on factor 1; dropped item 10 item 10: I am fearful of disapproval from others because of my sexual orientation. Factor 1: Internal Conflict	10.49 [1.93, 18.40]
Final: PPP = 0.181, BCFI = 0.996 [0.993, 1.000], BGH = 0.995 [0.991, 1.000], LOO-CV = 13,449.24	

N = 506 for all models that were estimated using non-informative priors and 3 chains of 6000 posterior samples each (i.e., 1000 burn-in and 5000 post-burn-in). Numbers in brackets are 90% highest density intervals. PPP = posterior predictive *p*-value; BCFI = Bayesian comparative fit index; BGH = Bayesian Gamma Hat; LOO-CV = leave-one-out cross-validation.

**Table 4:** Final Factor Structure of Perceived Internalized Sexual Stigma Measure (PrISSM): Standardized Loadings.

Item	F1: Internal Conflict [95% HDI]	F2: Disclosure Conflict [95% HDI]
I am angry because of my sexual orientation.	0.90 [0.87, 0.92]	
I hope my sexual orientation would change.	0.86 [0.83, 0.89]	
People that accept their sexual orientation frustrate me.	0.85 [0.82, 0.88]	
I have thought about committing suicide because of my sexual orientation.	0.77 [0.73, 0.81]	
My uncertainty of others’ possible reactions leads me to hide my sexual orientation.		0.84 [0.80, 0.87]
I fear that people will judge me for my sexual orientation.		0.82 [0.78, 0.86]
The thought of coming out in my workplace gives me anxiety.		0.75 [0.70, 0.79]
I am uncertain how others will react to my sexual orientation.		0.73 [0.68, 0.78]

N = 506 for model fit: PPP = 0.181, BCFI = 0.996 [0.993, 1.000], BGH = 0.995 [0.991, 1.000], LOO-CV = 13,449.24, with F1 & F2 correlation = 0.54 [0.46, 0.61]; numbers in brackets are 90% highest density intervals. All models estimated using non-informative priors and 3 chains of 6000 posterior samples each (i.e., 1000 burn-in and 5000 post-burn-in). PPP = posterior predictive *p*-value; BCFI = Bayesian comparative fit index; BGH = Bayesian Gamma Hat; LOO-CV = leave-one-out cross-validation.

**Table 5:** Study 2 Internal Consistency Estimates.

Factor/Latent Variable	Items	Coefficient Alpha [95% CI]	N
PrISSM (F1, F2)	8	0.89 [0.87, 0.90]	506
Internal Conflict (F1)	4	0.91 [0.89, 0.93]	506
Disclosure Conflict (F2)	4	0.86 [0.84, 0.89]	506
IHP	9	0.96 [0.96, 0.96]	506
IHP-R	5	0.95 [0.94, 0.96]	506
Agreeableness	6	0.71 [0.67, 0.75]	504
Conscientiousness	6	0.75 [0.72, 0.79]	504
Open-Mindedness	6	0.72 [0.68, 0.75]	505
Extraversion	6	0.66 [0.61, 0.71]	504
Negative Emotionality	6	0.81 [0.79, 0.84]	505

Confidence intervals are normal theory bootstrap with 1000 bootstrap samples.

**Table 6:** Study 2 Factor Correlations.

Factor	1	2	3	4
1. Internal Conflict	1			
2. Disclosure Conflict	0.48 †	1		
3. Internalized Homophobia (IHP)	0.89 †	0.51 †	1	
4. Revised Internalized Homophobia (IPH-R)	0.89 †	0.48 †	0.98 †	1
5. Agreeableness	−0.41 †	−0.24 †	−0.40 †	−0.40 †
6. Open-Mindedness	−0.51 †	−0.14 *	−0.52 †	−0.52 †
7. Conscientiousness	−0.22 †	−0.20 †	−0.23 †	−0.21 †
8. Negative Emotionality	0.10 *	0.34 †	0.12 *	0.09 *
9. Extraversion	0.08	−0.19 †	0.06	0.07
Mean	9.08	14.56	25.86	13.86
SD	6.04	5.80	16.98	9.76
Number of items	4	4	9	5

N = 504 for all correlations. * indicates *p* < 0.05; † indicates *p* < 0.001. Each factor is the sum (composite) of the corresponding items.

**Table 7:** Bayesian Measurement Invariance Models.

Model	PPP	BCFI [90% CrI]	BGH [90% CrI]	LOO-CV	Δ ELPD	Δ SE	RATIO
Lesbian Females (N = 118) v. Gay Males (N = 124)							
Configural	0.020	0.97 [0.96, 0.98]	0.98 [0.97, 0.99]	6439.27	N/A	N/A	N/A
Metric	0.024	0.97 [0.96, 0.99]	0.98 [0.97, 0.99]	6432.16	3.56	2.08	1.71
Scalar	0.018	0.97 [0.96, 0.98]	0.98 [0.97, 0.99]	6427.42	2.37	2.88	0.82
Male (N = 132) v. Female (N = 132) Bisexuals							
Configural	0.703	1.0 [0.99, 1.0]	1.0 [1.0, 1.0]	6898.82	N/A	N/A	N/A
Metric	0.751	1.0 [1.0, 1.0]	1.0 [1.0, 1.0]	6887.95	5.43	2.87	1.89
Scalar	0.730	1.0 [1.0, 1.0]	1.0 [1.0, 1.0]	6882.53	2.71	2.62	1.03
Lesbians-Gay (N = 242) v. Bisexuals (N = 264)							
Configural	0.240	0.99 [0.99, 1.0]	0.98 [0.98, 1.0]	13,271.40	N/A		
Metric	0.030	0.99 [0.98, 0.99]	0.99 [0.99, 1.0]	13,282.94	5.77	6.34	0.91
Scalar	0.003	0.98 [0.98, 0.99]	0.99 [0.99, 0.99]	13,291.85	4.46	5.26	0.85

Models estimated using non-informative priors and 3 chains of 6000 posterior samples each (i.e., 1000 burn-in and 5000 post-burn-in). PPP = posterior predictive *p*-value; BCFI = Bayesian comparative fit index; BGH = Bayesian Gamma Hat; LOO-CV = leave-one-out cross-validation; ELPD = expected log pointwise-predictive density.

## Data Availability

The raw data supporting the conclusions of this article will be made available by the authors on request.
